# Periodontitis and physical activity: a scoping review

**DOI:** 10.3389/froh.2026.1786143

**Published:** 2026-07-15

**Authors:** Jorge Contreras-León, José Rubén Herrera-Atoche, Bertha Arelly Carrillo-Ávila, Víctor Manuel Martínez-Aguilar, Jaime Díaz-Zúñiga, Samanta Melgar-Rodríguez

**Affiliations:** 1 Faculty of Dentistry, University of Chile, Santiago, Chile; 2 Faculty of Dentistry, Autonomous University of Yucatán, Mérida, México; 3 Department of Medicine, Faculty of Medicine, University of Atacama, Copiapó, Chile; 4 Conservative Dentistry Department, Faculty of Dentistry, University of Chile, Santiago, Chile

**Keywords:** fitness, metabolism, periodontal disease, periodontitis, physical activity, physical resistance

## Abstract

Periodontitis is a chronic inflammatory pathology with a high prevalence in the population, and is associated with the chronic low-grade inflammatory phenotype (CLIP). Also, a sedentary lifestyle contributes to CLIP development. Physical exercise is considered a highly relevant activity for the prevention of chronic diseases. Though the evidence suggest an association between periodontitis and physical activity performance, it is currently unknown whether periodontitis can affect physical performance or how physical activity could ameliorates periodontitis. This is a scoping review that was carried out based on primary research studies, clinical trials, and observational studies identified in PubMed, SciELO and LILACS, Google Scholar and Cochrane Library, published since 2000, which will have the criteria for diagnosing periodontitis and evaluating physical exercise. Based on 23 selected studies we analyzed how in both human and animals studies the exercise could play a protective role for inflammation or bone destruction. In fact, the physical exercise attenuates the inflammation and alveolar bone loss in rodents and humans. However, the widely heterogeneity of the population, the wide variety of methods used to evaluate the exercise, among other variables prevent this data from providing a conclusive answer. Thus, randomized clinical trials are needed to determine the true effect of exercise on reducing inflammation and periodontal bone loss.

## Introduction

Periodontitis is an inflammatory chronic non-communicable disease (CNCD) caused by the dysbiosis of oral microbiota, that affect the supporting tissues of the teeth (1). The susceptibility to periodontitis onset is influenced by genetic, epigenetic, and environmental factors, that affect both the microbiota stability or the immune response (2). Besides, in 291,170 individuals in 37 countries sowed that the prevalence of periodontitis in the 35–44 years-old population is 40%, which indicates a significant increase in the disease's incidence with age (3, 4). Recently, periodontitis has been increasingly linked to the onset and progression of various CNCD. These include type II diabetes, dyslipidemia, fat liver, and even Alzheimer's disease. Furthermore, periodontitis can contribute to a decrease in glucose uptake and peripheral insulin resistance, further exacerbating metabolic disorders (5, 6).

In humans, skeletal muscle is the primary tissue responsible for glucose uptake in both the postprandial state and under conditions of euglycemic hyperinsulinemia (7). Physical activity is defined as any bodily movement produced by skeletal muscles that results in energy expenditure. Meanwhile, exercise is not synonymous of physical activity, but rather a subcategory of it (8). Health-oriented physical fitness is generally described as having four key components: body composition, aerobic capacity, musculoskeletal capacity, and flexibility. Musculoskeletal capacity is the ability of the skeletal and muscular systems to perform work that requires muscular strength and endurance (9). Regular exercise is crucial for preventing lifestyle-related diseases, and inactivity is a major contributor to CNCD like obesity (10–12). Research suggests that physically active individuals have a lower risk of developing periodontitis compared to those with sedentary lifestyle (10, 11, 13). Furthermore, some studies reported that poor oral health impacts physical activity and performance: oral pain (29.9%), difficulty participating in normal training and competition (9.0%), performance affected (5.8%), and reduction in training volume (3.8%) (14, 15).

Otherwise, the sedentarism is a several public health problem that contributes to chronic lowgrade inflammatory phenotype (CLIP) and visceral fat accumulation, both of which are linked to an increased risk of various CNCDs (16–19). Thus, both skeletal muscle activity and sedentarism are key factors that influence the development and prevalence of metabolic, cardiac, degenerative and immune diseases (20–23). Observational studies consistently demonstrate an inverse association between physical activity and inflammation.

Individuals who engage in more frequent and intense physical activity, including both leisure and non-leisure activities, tend to have lower concentrations of inflammatory biomarkers in their bodies (24, 25). Other studies demonstrate an inverse relationship between physical activity and inflammatory markers like C-reactive protein (CRP), even when the activity is at a low intensity (26–28). Exercise indeed promotes anti-inflammatory signals in the body. Specifically, it reduces the expression of Toll-like receptors (TLR)-2 and TLR4 on immune cells, which are involved in the inflammatory response. Furthermore, exercise decreases the infiltration of macrophages into adipose tissue, and improves blood and nutrient supply of adipocytes in the visceral fat mass, which can reduce inflammation (29–31). Some circulatory biomarkers that are released acutely and contribute to these anti-inflammatory signals are IL-6 and IL-10 (32, 33). In obese rats, experimental evidence suggests that exercise can decrease the production of pro-inflammatory cytokines such as TNF-α, IL-1β and IL-12, while increasing the levels of the anti-inflammatory cytokine IL-10 within visceral adipose tissue (34–36). This shift in cytokine profile is believed to be a beneficial in mitigating inflammation associated with obesity and its related metabolic disorders.

It is of public health interest to manage periodontitis, a common inflammatory disease linked to other chronic conditions, by reducing its pro-inflammatory component through strategies that don't solely rely on clinical or pharmaceutical interventions. Regular physical activity may offer a behavioral strategy to limit CLIP and significantly reduce health care costs by reducing the number of hospitalizations, medical visits, and need for medications (37, 38). Otherwise, given the relationship between physical activity and inflammation, and considering the proinflammatory markers that are involved in both periodontitis and muscle metabolism, it is plausible that the CLIP triggered by periodontitis can also influence physical condition (25, 39–41). However, there is still very limited evidence to suggest an association between periodontitis and decreased physical performance.

## Methods

### Review question

What evidence exists to demonstrate that physical exercise plays a protective role against the development or progression of periodontitis?

### Inclusion criteria

#### Participants

We include studies carried out in both humans or animals, in which the population was clearly identified. We selected studies conducted in humans over 18 years-old, regardless of distribution by sex, socioeconomic level or ethnicity (participants). Also, we selected studies conducted in young adult rodents ≥6 weeks of age (participants).

#### Concept

Te selected studies had to describe the presence of physical exercise or physical activity in both humans and animal studies. In the experimental studies we include studies with 1) swimming; 2) forced treadmill running and; 3) voluntary treadmill running. In the human studies we selected articles where people were subjected to voluntary exercise, or where the type of exercise people performed was evaluated. In addition, we also selected the studies in animal models where periodontitis was induced or in humans where periodontitis was diagnosed.

#### Context

In all the selected studies we identified the laboratory or university associated with the experimental study. For studies conducted on humans, we recorded the university or health center where the study was performed. In all studies, we identified the country of origin of the laboratories, universities, or health centers.

### Searching for the evidence

The present scoping review was carried out following the Joanna Briggs Institute guidelines. The full protocol of data search, selection, extraction, and analysis of the information was discussed among all the authors, who entered the information into a dynamic drive. Studies published since 2000 were selected, without restriction on publication status or language. If an article was written in a language different from English, Spanish or Portuguese, a qualified translator was consulted. The core databases were MEDLINE, Embase, and Central. Selected articles were included in a full-text analysis, and non-selected articles were included into a table indicating reasons for exclusion. Primary research studies, randomized or nonrandomized clinical trials, case-control and cohort studies, and experimental researches were included. The grey literature source were articles, and theses found in google scholar. Systematic reviews with or without meta-analysis were excluded from the analysis, as they are not a primary source of information. Also, we revised the clinical trials registries (www.clinicaltrials.gov) in order to detect clinical trials not found in the primary resources. The protocol was registered in Open Science Framework with the DOI: 10.17605/OSF.IO/6JV74 (https://osf.io/6jv74/overview). The search strategy was carried out using the MeSH terms “periodontitis”, “exercise”, “physical activity”, “physical fitness”, and “physical performance” in the Medline database and adapted for the other databases of data. All the search strategy forms are in the Supplementary Appendix file 1.

#### Selection of evidence

The selection of studies was performed independently and in duplicate by two reviewers (JCL and JD-Z). In cases where there were doubts regarding the inclusion of a study, a third reviewer was responsible for deciding its inclusion or exclusion (SM-R). The articles obtained from each database were listed identifying the authors, age, title, journal, volume, pages, and doi. After that, the databases were matched and duplicates were subsequently removed. Titles and abstracts were then evaluated, and articles not associated to the review question were eliminated. Finally, the studies that met the criteria were downloaded for full-text analysis.

Data extraction followed a structured approach; a template was created to extract the key features of each included document (Supplementary Appendix 2). The data elements included in the template were the first author's name, year of publication, study design, study population (number, sex, and age), group definition, intervention or exposure, type of exercise or physical activity, periodontal diagnostic criteria, results focused on the effect of exercise on bone resorption caused by periodontitis, and the origin. The Cochrane Handbook was used as a guide for the data collection process (42).

### Outcome measures

To evaluate the relationship between physical exercise/activity and periodontitis, the primary outcome was to determine if the physical exercise reduce the periodontitis risk. As a secondary outcome we selected studies that demonstrate the protective effect of physical exercise on bone loss in patients with periodontitis. The contribution of the selected articles to each of the results is summarized in Table 1.

**Table 1:** Population, concept, and context table of experimental studies.

		Population		Concept			Contex
	Author and year	Specie	Total number	Physical exercise evaluation	Periodontal diagnostic criteria	Results	Origin
1	Andrade et al., 2017	Wistar rats	24	Swimming for 60 min/day for 8 weeks	Periodontitis induced by ligature	The exercise training attenuated bone loss and epithelial attachment loss levels of rats affected with periodontitis (*p* < 0.05). Animals trained affected with periodontitis presented a lower expression of TNF-α and increased levels of IL-10 in periodontal tissues (*p* < 0.05).	Brazil
2	Andrade et al., 2018	Wistar rats	40	Swimming for 60 min/day for 8 weeks	Periodontitis induced by ligature	In rats with periodontitis the TNF-a levels were 8.66 pg/mL, and decrease with physical training to 6.86 pg/mL. Also, the levels of IL-10 in rats with periodontitis were 10.77 pg/mL and increase with the exercise to 13.69 pg/mL. The rats with physical training show an alveolar bone high of 0.94 mm, meanwhile the rats with periodontitis presented a loss of 1.13 mm. The bone loss decrease in presence of physical exercise to 1.02 mm. All the comparisons were significative with a pvalue <0.05.	Brazil
3	Bertolini et al., 2022	Wistar rats	32	Treadmill training	Periodontitis induced by ligature	The total volume of physical activity and the speed of activity decreased over the seven days after ligation placement (*p* < 0.05). Ligature placement resulted in significant bone resorption and increased expression of RANKL, IL-1β, IL-6, and TNF-α. Exercise reduced bone loss (*p* < 0.05) and the expression of TNF-α and IL-1β, while it increased the expression of OPG.	Brazil
4	Bortolini et al., 2019	Wistar Rats	24	Swimming for 4 weeks, with progressive increase in time	Periodontitis induced by ligature	It was found that bone loss in animals in the exercise group was significantly lower (61.7 ± 2.2; *p* < 0.05) than the rats affected with periodontitis (84.5 ± 1.2; *p* < 0.05). In terms of the number of osteoblasts and osteocytes the group with exercise had more cells (19.4 and 29.3, respectively) compare with the rats affected with periodontitis (11.0, and 17.3, respectively).	Brazil
5	Da Silva et al., 2015	Wistar Rats	24	Swimming for 60 min/day, for 8 weeks.	Periodontitis induced by ligature	The results showed that the parameters with significant differences were the levels of red blood cells (erythrocytes, hemoglobin and hematocrit) and the lymphocyte count, where the exercise increase the values in rats with periodontitis, compared with those without exercise (*p* < 0.05).	Brazil
6	de Souza et al., 2020	Wistar Rats	24	Treadmill training (5 times/week for 8 weeks).	Periodontitis induced by ligature	The exercised rats had lower leukocytes counts compared with exercised rats with periodontitis. The levels of TNF-a were higher and IL-10 were lower in trained rats with periodontitis compared with rats exercised without disease. After the cryoinjuries, the rats trained and with periodontitis had more inflammation in *tibia's* anterior, and *gastrocnemius* muscles compared with rats without periodontitis.	Brazil
7	Hayashi et al., 2022	Wistar Rats	20	Treadmill training (25 m/min, 30 min, 4 days/week, 4 weeks)	Oral gavage of *Porphyromonas ginigivalis*	The rats affected with periodontitis had less time to running exhaustion, which demonstrate that the oral gavage with *P. gingivalis* decrease the performance in the physical test.	Japan

## Results

### Search results

**Figure 1:**
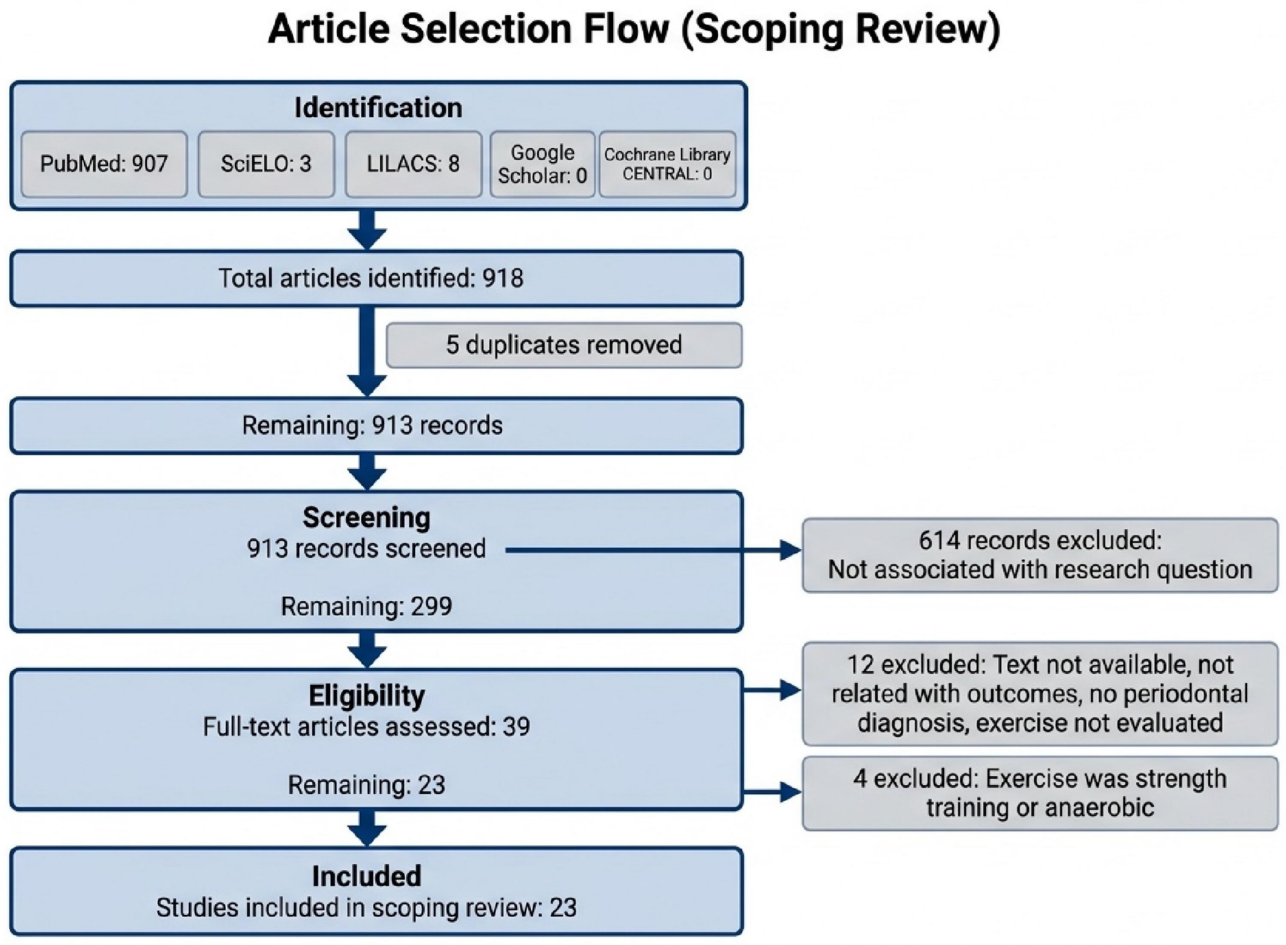
Articles selection diagram. The Figure represents the summary of the article search. From a universe of 918 articles, 653 passed the first review of abstract and title. Subsequently, the articles not related with outcomes and another reasons were eliminated to remain with 39 articles that were reviewed in full text. Finally, 26 articles were selected to prepare this scoping review.

A total of 918 articles were obtained from the search carried out in the databases PubMed (907), SciELO (3), LILACS (8), Google Scholar (0) and Cochrane Library CENTRAL (0) in the identification stage. Of these studies, 5 were duplicates, so 913 were selected for abstract reading. A total of 614 studies whose objective was not associated with the research question were excluded from this process. After this, 39 articles were analyzed for eligibility by reading the full text, of which 12 were excluded for text not available, not related with outcomes, articles without periodontal diagnosis or exercise not evaluated. Finally, 4 studies were excluded because the exercise was either strength training or completely anaerobic (Figure 1). Finally, 23 studies were included in the scoping review (Supplementary Table 1). The reason for exclusion of the remaining articles is found in Supplementary Table 2. The studies included in the selection corresponded to 7 experimental studies (Table 1) and 16 studies in humans (Table 2), of which 8 correspond to descriptive studies, 2 are observational studies, 1 cases and controls, 4 cohorts, and 1 clinical trial. Supplementary Table 3 identifies the outcome to which each study selected for the objective of this Scoping Review contributes.

**Table 2:** Population, concept, and context table of human studies.

			Population	Concept			Context
	Author and year	Study design	Total number	Physical exercise evaluation	Periodontal diagnostic criteria	Results	Origin
1	Alkan et al, 2020	Cohort	39	Aerobic exercise program for 12 weeks	Plaque index, gingival index, probing pocket depth, bleeding on probing, gingival recession, and clinical attachment level.	Probing pocket depth decrease in patients with periodontitis after the training program (3.05 mm to 2.90 mm, *p* < 0.05). Also, the clinical attachment level loss decrease after the exercise in patients with periodontitis (3.65 mm to 3.47 mm, *p* < 0.05).	Turkey
2	Al-Zahrani et al, 2005	Descriptive	12,110	Self-report and questionnaire identifying: walking a mile or more at a time without stopping, jogging or running, bike riding, aerobic dancing, dancing, swimming, calisthenics, garden or yard work.	Pocket probing depth, and clinical attachment level.	People who maintained a normal weight, a recommended level of exercise, and a high-quality diet were 40% lower chance of developing periodontitis. It cannot be determined how the exercise protect against periodontitis, nevertheless, the presence of 1, 2 or 3 of the healthy behavior (normal weight, exercise or high quality diet) had an *odds ratio* of 0.84, 0.71, and 0.60.	United States
3	Al-Zahrani et al, 2005	Descriptive	2,521	Self-report and questionnaire identifying: walking a mile or more at a time without stopping, jogging or running, bike riding, aerobic dancing, dancing, swimming, calisthenics, garden or yard work.	Pocket probing depth, and clinical attachment level.	Engaging in the recommended level of physical activity was significantly associated with a lower prevalence of periodontitis (OR = 0.58, 95% CI, 0.35–0.96). While people with physical activity had a 13% of prevalence of periodontitis, those with inactive behavior had a prevalence of 20% (*p* = 0.001).	United States
4	Bawadi et al, 2011	Descriptive	340	Self-report with International Physical Activity Questionnaire (heavy lifting, digging, aerobics, fast bicycling)	Plaque index, gingival index, probing pocket depth, and clinical attachment loss.	More physically active individuals had significantly lower mean of plaque index (1.42 v/s 1.32), gingival index (1.61 v/s 1.36), clinical attachment loss (2.69 v/s 2.18), and percentage of sites with CAL ≥3 mm (19.36 v/s 8.83) compared to people with a low level of physical activity. In multivariate analysis, a low level of physical activity and a poor diet (diets with a healthy eating index <50 points) were significantly associated with increased odds of periodontitis.	Jordan
5	Holtfreter et al, 2021	Case-controls	4,078	Cardiopulmonary exercise was tested quantifying the peak oxygen uptake, oxygen uptake at anaerobic threshold, the minute ventilation changes, the peak oxygen pulse, and exercise duration.	Mean probing pocket depth, clinical attachment level, and number of teeth.	The people with higher probing pocket depth (3.7 mm) had a decrease in the oxygen consumption at anaerobic threshold (1,89 mL/min), in the slope of ventilation efficiency to remove carbon dioxide (23.9 mL/min/kg), and in the maximum oxygen pulse(27.53), when compared with people with less depth (1.6 mm) who had a better performance. People with more clinical attachment loss had a decrease in both oxygen consumption and exercise duration (1,849 mL/min, and 8.7 min, respectively) when compare with people with less clinical attachment loss (2,045 mL/min, and 9.3 min).	Germany
6	Hoppe et al, 2017	Observatio nal cross-sectional study	112	Physical Fitness Test. Evaluation of physical strength and cardiorespiratory fitness	Probing pocket depth, clinical attachment level.	Higher probing pocket depth, and clinical attachment loss were associated with poor physical fitness in males (*p* < 0.05). Also, the regular exercise had an odds ratio of 1.78.	Brazil
7	Hwang et al, 2022	Crosssectional validation and reliability study	13	International Physical Activity Questionnaires	Community Periodontal Index, probing depth	Each of the healthy lifestyle practices like diet quality, physical activity, and normal body weight were significantly associated with periodontal diseases: OR: 1.32, 95% CI: 1.13–1.55; OR: 1.16, 95% CI: 1.04–1.30; OR: 1.26, 95% CI:.14–1.40, respectively. In particular, having poor healthy lifestyles practices were identified as a risk factor for periodontal diseases: OR: 1.54, 95% CI: 1.10–2.15.	Korea
8	Marruganti et al, 2023	Observatio nal, populationbasedcross- sectional study	11	Global Physical Activity Questionnaires to assess Leisure Time Physical Activity and Occupational Physical Activity.	Probing pocket depth and clinical attachment level	Multiple regression analyzes identified high leisure time physical activity as a protective indicator for periodontitis (OR = 0.81, 95% CI, 0.72–0.92), while high occupational physical activity turned out to be a significant risk indicator (OR = 1.16; 95% CI: 1.04–1.30). The combination of low leisure time physical activity/high occupational physical activity showed a cumulative independent association with periodontitis (OR = 1.47; 95% CI: 1.26–1.72). Furthermore, both high leisure time physical activity (OR = 0.72; 95% CI: 0.58–0.90) and high occupational physical activity (OR = 1.29; 95% CI: 1.09–1.53) were significantly associated with stronger estimates of severe periodontitis. The same was observed for the combination of low leisure time physical activity/high occupational physical activity (OR = 1.66; 95% CI: 1.29–2.15).	Spain
9	Mendoza-Núñez et al, 2014	Cohort	61	Tai Chi sessions of 60 min, 5 days a week for 60 min/session, for 6 months.	Periodontal disease index.	In people who performed the Tai Chi program the cytokine IL-1β (783.62–624.9, *p* < 0.01), IL-6 (18.66–4.76, *p* < 0.05) decrease significantly after 6 months. Also, the periodontal disease index decrease after 6 months of Tai Chi (3.626 mm to 3.281 mm, *p* < 0.05).	Mexico
10	Merchant et al, 2003	Reliability and validity study	39,461	Questionnaires inducing walking, hiking, jogging, running, bicycling, using stationary bicycle, swimming, playing tennis, squash)	Self-report of periodontal disease and radiographic exam.	The risk of periodontitis decreased by 3% for every 10 metabolic equivalents increase in average physical activity after adjusting for age, smoking, diabetes, BMI, alcohol consumption, and total calories (RR = 0.97; 95% CI: 0.95–0.99). Compared with men in the lowest quintile of physical activity, those in the highest quintile had a 13% lower risk of periodontitis (RR = 0.87, 95% CI: 0.76–1.01, p, trend test = 0.02). In a subsample of men with radiographs (*n* = 137), those physically active had less mean bone loss (beta = −0.29, *p* value = 0.03) after multivariable adjustment compared with those inactive.	United States
11	Merle et al, 2022	Cohort	85	Maximum aerobic capacity (VO_2_max), sports performance tests with incremental running or cycle ergometer exercises, resting heart rate, heart rate, lactate and power.	Papillary bleeding index, periodontal screening index.	Athletes with periodontal screening index ≥3 had lower VO_2_max values (55.9 ± 6.7 mL/min/kg vs. 59.3 ± 7.0 mL/min/kg; *p* = 0.03) In exercise tests the athletes with papillary bleeding index < 0.42 achieved a higher relative maximal load on the cycle ergometer (5.0 ± 0.5 W/kg vs.4.4 ± 0.3 W/kg, *p* = 0.03).	Germany
12	Omori et al, 2018	Randomiz ed, control, parallelgroup clinical trial	50	Resistance training (exercise volumen around 170–190 kcal/session), or aerobic training in a cycling session (20–40 min at 60%–85% VO_2_ Max).	Dental questionnaire, oral exam (teeth number, dental mobility, probing pocket depth, bleeding on probing), saliva sampling.	In the exercise intervention group, the number of teeth with PPD ≥4 mm decreased significantly from 14.4% to 5.6% (*p* < 0.001), and the number of teeth with bleeding on probing decreased significantly from 39.8% to 14.4% (*p* < 0.001). Copy counts of *Porphyromonas gingivalis* increased significantly (*p* = 0.001).	Japan
13	Oliveira et al, 2015	Crosssectional observational	111	Physical Fitness Test: push-ups, pull-ups, sit-up, running.	Probing pocket depth, clinical attachment loss, and bleeding on probing.	Individuals presenting at least one tooth with CAL ≥4 mm had significantly lower physical fitness test scores (277.8 ± 23.6 points) compared to those without this status (285.9 ± 20.2 points). A 1 mm increase in PD or CAL significantly decreased the chance of achieving the highest physical fitness test score by 69% or 75%, respectively.	Brazil
14	Park et al, 2015	Cohort	53	Aerobic training 2 h a day, weight training 3 h a day, for 4 weeks	Plaque index, gingival index, bleeding on probing, probing pocket depth.	After the training program, no changes were detected in the periodontal parameters in both healthy an obese people. Also, in both groups, the levels of IL-1β decrease significantly (obese people from 96.8 to 38.5, *p* < 0.001; healthy people from 66.6 to 56.5, *p* < 0.05).	Korea
15	Sanders et al, 2009	Case- control	751	Self-administered questionnaire of the leisure-time physical activity	Probing pocket depth, recession, and clinical attachment level.	Those subjects who met a prescribed threshold for leisure-time physical activity had lower adjusted odds of elevated IL-1β than less active adults. Physical activity was not associated with periodontitis. Periodontitis modified the association between physical activity levels and detectable CRP. Increasing quartiles of physical activity were associated with decreasing probability of detectable CRP, but the effect was limited to periodontitis cases and was not evident among controls.	Australia
16	Wernicke et al, 2021	Randomiz ed Controlled Trial	37	Resistance exercises, aerobic training or a combination of both, 2 times a week for 6 months	Probing pocket depth, bleeding on probing, and plaque index.	Physical activity showed a positive effect on periodontal health. Both bleeding on probing (*p* = 0.005) and periodontitis severity (*p* = 0.001) were significantly reduced in the intervention group compared to the control group. The probing pocket depth decrease from 2.27 mm to 1.38 mm after the exercise routine.	Germany

### Review findings

#### Analysis and presentation of the results in experimental models

**Figure 2:**
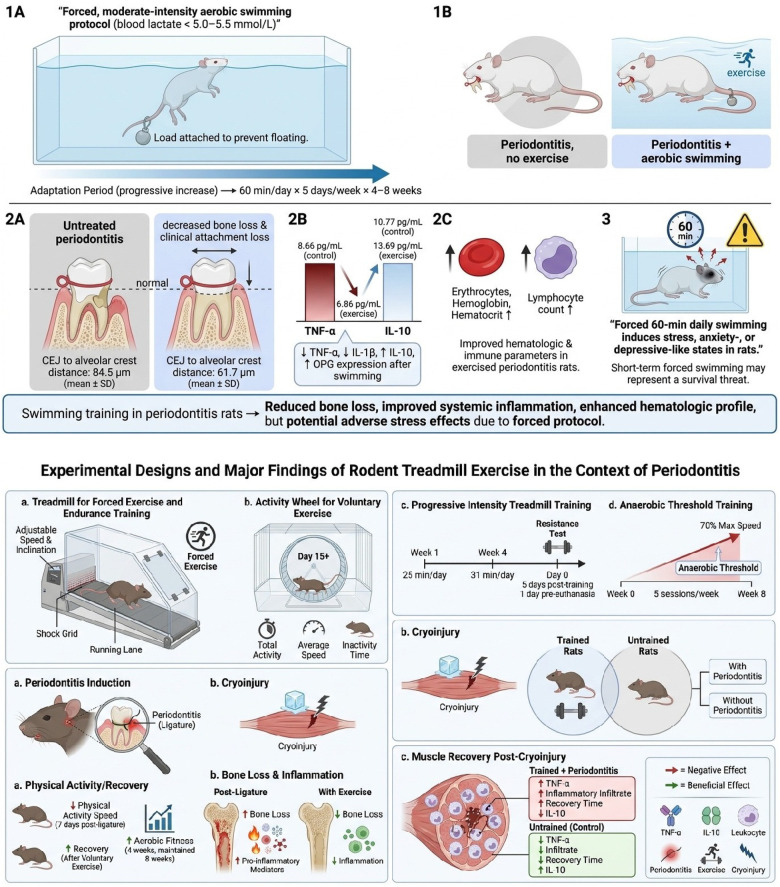
A Summary of principal findings in aerobic swimming exercise in rats. **(1A)** Represents the exercise models, **(1B)** show the experimental and control groups. The **(2A)** section represent the main clinical findings according bone loss in rats, **(2B)** represents a summary of the pro-inflammatory mediators, and **(2C)** the immune cells changes. The item 3 represents the main findings of the experimental studies. **(2B)** Summary of principal findings in treadmill exercise in rats. The figure represents a summary of Table 1. In the upper-left scheme is represented the exercise design. In the lower-left scheme the main findings are represented grouped into animals in which periodontitis was induced and those in which cryolesion was induced to observe the recovery time. On the right schemes, other studies conducted with treadmills and endurance tests are also shown. The figure highlights the ability of animals without periodontitis to recover from cryoinjury with exercise.

Seven experimental studies using male Wistar rats from 5 to 8 weeks of age were identified where ligatures are placed around the upper molars to mimic human disease progression (43–49) (Table 1, Figures 2A,B). In those articles, the evaluation of physical activity used two different aerobic exercises: aerobic swimming (43–46) and free walking on a treadmill (47–49).

##### Aerobic swimming

The aerobic swimming in animals is a specialized, forced exercise protocol designed to increase oxygen consumption for energy production and cardiovascular fitness. It is typically defined as continuous, moderate-intensity exercise that keeps blood lactate levels below 5.0–5.5 mmol/L. Unlike human swimming, rats are forced to swim vertically in deep tanks and often carry a small attached load to prevent them from floating and ensure a constant effort.

The animals subjected to aerobic swimming went through an adaptation period to reduce stress to the aquatic environment, without promoting training. The following week the animals began swimming sessions with progressive increments of time, until reaching 60 min of daily swimming without load. This training was replicated 5 times a week, for 4 weeks (48) or 8 weeks (Figure 2A) (43–45).

All the animals were induced periodontitis by ligature. Among the most important results, the animals with periodontitis and which underwent to swimming training show a decrease in the bone loss and clinical attachment loss (44–46). Also, the swimming training decrease the TNF-α serum levels in groups with periodontitis from 8.66 pg/mL to 6.86, and increase the levels of IL-10 from 10.77 pg/mL to 13.69 pg/mL (44, 45). The article by Bertolini et al. points out that swimming decreases the expression of TNF-α, and IL-1β, reduced alveolar bone resorption, and increased the expression of OPG (46). In fact, the exercise group was a distance between the cementum-enamel junction to the alveolar crest significantly lower (61.7 µm ± 2.2) than the rats affected with periodontitis and without swimming (84.5 µm ± 1.2; *p* < 0.05) (46). Moreover, da Silva et al., showed that the parameters with significant differences in the exercised rats were the levels of red blood cells (erythrocytes, hemoglobin and hematocrit) and the lymphocyte count, where the exercise increase the values in rats with periodontitis, compared with those without exercise (*p* < 0.05). Nevertheless, the 4 aforementioned studies were performed in a swimming program for 60 min daily. It is noteworthy to mention, due to subjecting rats to a daily 60-min swimming task—specifically in an inescapable, forced-swim scenario—is a highly effective method to induce significant stress, anxiety-like behaviors, or depressive-like states. In this context, the design of shortterm swimming could be interpreted by the animal as a survival threat (50).

##### Treadmill training

The treadmills are specialized devices designed for forced exercise, endurance training, or metabolic studies in rodents. The treadmills typically feature adjustable speeds, inclinations, and shock grids to motivate movement. In the selected studies, the animals subjected to free walking on a treadmill had different evaluation methods (Table 1, Figure 2B). In one study, rats were given access to activity wheels starting on day 15 to allow for voluntary physical activity. This setup enabled the researchers to measure the total volume of activity, average speed and the time of inactivity for each rat (48). In a second study, rats were exercised on a treadmill with progressive intensity for 25 min daily during the first week, increasing to 31 min a day by the fourth week. This progressive overload training method involves gradually increasing the difficulty of the workout over time. Besides, a resistance test was performed 5 days after the last training and one day before euthanasia (49). In a final treadmill study trained rats for 8 weeks, starting 30 days in, with 5 sessions per week, to gradually increase their speed until they reached 70% of their maximum speed to determine their anaerobic threshold. This progressive endurance exercise was designed to improve aerobic performance and allow for physiological adaptations to be studied, which has been shown to increase fitness within 4 weeks and maintain it for the duration of the study (47).

Rats affected by periodontitis induced by ligature showed a temporary reduction in physical activity speed after 7-days of ligature installation (48). Though the installation of the ligature produce bone loss and an increase in the pro-inflammatory mediators, the exercise reduced them significantly. This recovery could be linked to the rats' voluntary physical activity and its positive effect on reducing bone loss and inflammation (48). In the other study, rats were trained in a treadmill, and after periodontitis induction some cryoinjuries were induced in order to determine the muscle recovery in presence/absence of periodontitis and exercise (47). The high-intensity exercise causes muscle damage, which prompts an increase in leukocytes within the damaged muscle tissue to initiate a repair response (47). Indeed, rats with periodontitis had higher levels of TNF-α and lower levels of IL-10 compared to rats without periodontitis. After injury, trained rats had a longer recovery time compared to untrained rats, and rats with periodontitis had greater inflammatory infiltrate in their muscles compared to rats without periodontitis (47).

### Analysis and presentation of the results in human studies

**Figure 3:**
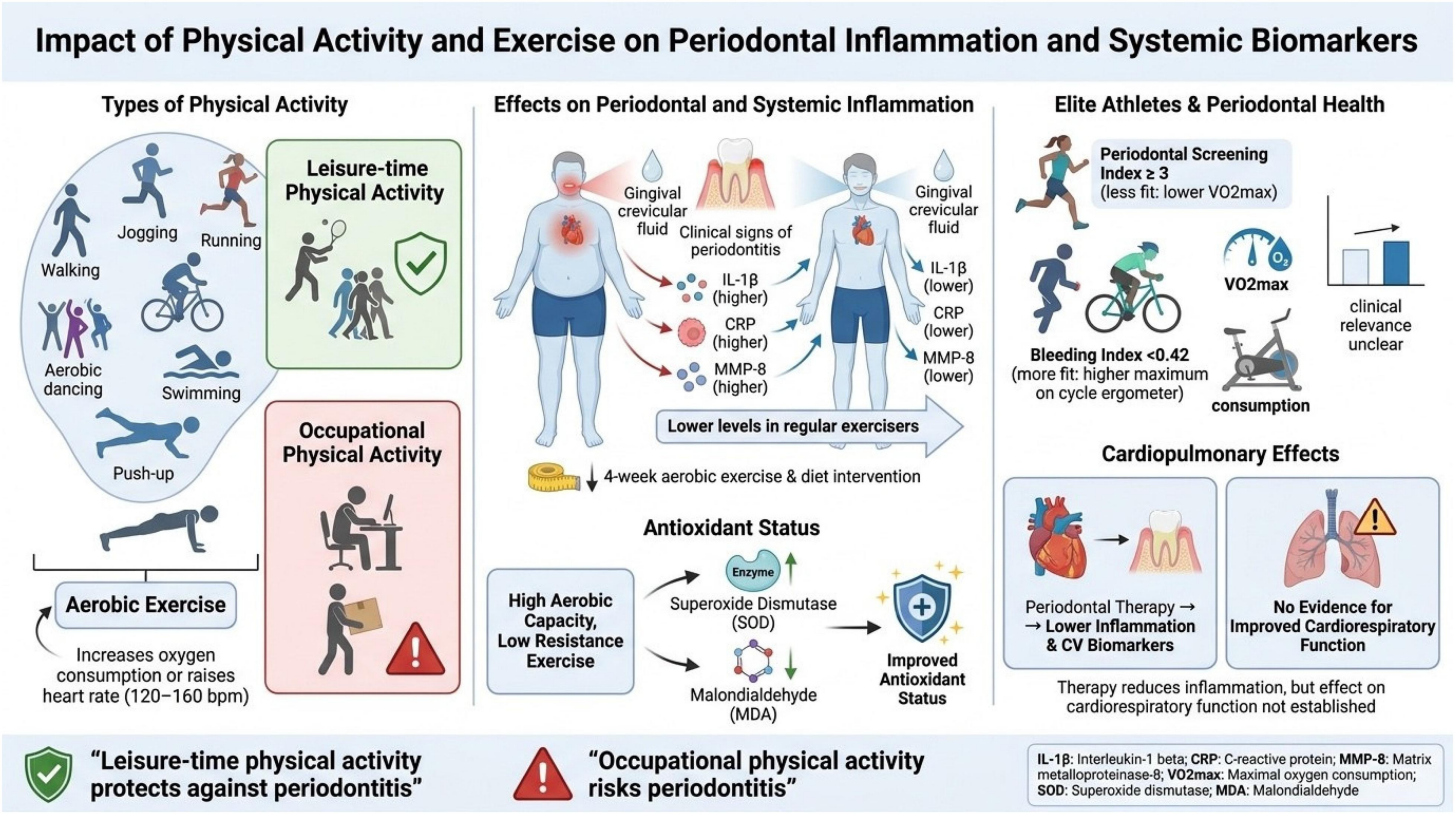
Summary of descriptive studies in humans. The figure shows the types of physical exercise performed on the selected studies. Also, it shows the effect of the exercise on periodontal and systemic inflammation, and the cardiorespiratory function.

Within the search, 16 studies carried out in humans were selected, of which 8 correspond to descriptive studies (10, 11, 51–56), 2 observational (57, 58), 1 cases and controls (38), 4 cohorts (59–62), and 1 clinical trial (63) (Table 2, Figure 3). The study populations consists of individuals over 18 years of age who received a periodontal diagnosis through an intraoral examination that uses periodontal parameters and indices. The evaluation of physical exercise was carried out through various methods of physical activity, where 7 studies evaluated it through resistance and/or aerobic training (53, 54, 57, 58, 60–63), and 7 studies applied validated questionnaires or self-reports (10, 11, 38, 51, 52, 55, 56, 64).

#### Resistance or aerobic training and periodontitis

The aerobic exercise programs were varied, including different types of exercises capable of increasing oxygen consumption or raising the heart rate to a range of 120–160 beats per min. The exercises evaluated included: walking, jogging, running, bike riding, aerobic dancing, swimming, calisthenics, among other. Also, the leisure-time physical activity refers to body movements performed during leisure time—sports, exercise, and recreational walking—, and occupational physical activity which corresponds to movements performed as part of professional tasks—long-lasting static loads and repetitive work postures (65–67).

Several studies reported that physical exercise decreased the IL-1β serum levels in patients affected by periodontitis (38, 48, 59, 60) (Figure 3). People who exercise regularly tend to have lower levels of pro-inflammatory markers like IL-1β and CRP in their gingival crevicular fluid, and also show fewer clinical signs of periodontitis (38, 59). Interestingly, the weight control through aerobic exercise and diet restriction for one month could both reduce the levels of biomarkers of periodontal inflammation and weight. A 4-week intervention can lower levels of periodontal biomarkers like MMP-8 in the gingival crevicular fluid, as well as reduce measures of obesity such as BMI and waist circumference (60). Furthermore, if we analyze a type of exercise with less physical resistance but higher aerobic capacity, the researches indicates that can improve the total antioxidant status. The studies shown that the less physical resistance exercise can increase the activity of enzymes like superoxide dismutase and reduce markers of oxidative stress like malondialdehyde (68–70).

Related with the pro-inflammatory cytokines, only one study quantified the concentrations, demonstrating that after the exercise a slight decrease in serum levels is observed in patients with periodontitis (59). These findings are consistent with other studies which demonstrate that physical training modulates IL-6 levels in individuals with CLIP (71).

Another recent study in elite athletes noted that athletes with a periodontal screening index ≥3 had lower VO_2_max values, and athletes with a bleeding index <0.42 achieved a highest relative maximum on the cycle ergometer (62). Though there are consistent cross-sectional associations of probing depth with VO_2_max peak and exercise duration, the effects are probably not clinically relevant as it is not clear whether periodontal therapy would have clinical effects on cardiopulmonary function (54). Despite periodontal therapy have beneficial effects on both inflammation and cardiovascular health biomarkers, there is not enough evidence to support the relevance of periodontal treatment on cardiorespiratory function (72, 73). Eventhough leisure-time physical activity represents a protective indicator of periodontitis, occupational physical activity is a periodontitis risk indicator (56).

#### Self-report exercise and the association with periodontitis

Six studies evaluated the effects of exercise on periodontal health in humans (10, 11, 51, 52, 55, 56) (Figure 3B). In fact, people who performed the recommended level of exercise and had a high-quality diet had 40% less probability to have periodontitis compared to people who did not maintain any of these healthy behaviors (10, 11, 55). Also, 3 studies observed that probing depth, clinical attachment level, chronic pro-inflammatory cytokines load, and severity of periodontitis were associated with low physical fitness (52, 58, 63).

Based on the data, we design the RR or OR values to a better comprehension of the extracted information. According the self-reported activity and the periodontal examination, the different studies concluded that physical activity can modify the periodontitis risk. Among the most striking findings is that occupational or work activity is a risk factor for developing periodontitis (OR = 1.16). If we combine low leisure time with high occupational/work activity, the risk of developing more severe periodontitis increases 1.66 times. The main findings and their clinical relevance are represented in Table 3.

**Table 3:** Relative risk and odd ratio of the selected studies.

Category/comparison	Relative risk (RR)	Statistical confidence/significance	Analysis
Every 10 MET increase in average physical activity	RR = 0.97	95% CI: 0.95–0.99	A statistically significant 3% risk reduction for every 10 MET increase.
Highest vs. lowest quintile of physical activity (Men)	RR = 0.87	95% CI: 0.76–1.01(ptrend = 0.02)	A 13% lower risk for those in the highest tier of activity. The trend across quintiles is statistically significant.

Category/risk factor	Odds ratio (OR)	95% confidence interval	Clinical interpretation
Poor healthy lifestyle Practices (overall)	OR = 1.54	1.10–2.15	54% higher odds of developing periodontal diseases if overall lifestyle choices are poor.
Poor diet quality (HEI <50 points)	OR = 1.32	1.13–1.55	A low-quality diet independently increases disease odds by 32%.
Obesity (overweight/abnormal body weight)	OR = 1.26	1.14–1.40	Lacking a normal body weight increases disease odds by 26%.
Low physical activity (general)	OR = 1.16	1.04–1.30	General physical inactivity independently elevates disease odds by 16%.
High leisure-time physical activity	OR = 0.81	0.72–0.92	Protective Indicator: Reduces the odds of periodontitis by 19%.
High occupational physical activity	OR = 1.16	1.161.04–1.30	Risk Indicator: Heavy physical labor at work increases disease odds by 16%.
Low leisure + high occupational activity	OR = 1.47	1.26–1.72	Cumulative Risk: Combining a sedentary home life with heavy manual labor increases disease odds by 47%
Regular exercise routine (general)	OR = 1.78	Not specified in text	Individuals who exercise regularly have 1.78 times higher odds of maintaining health/meeting protective thresholds.

In general terms, higher levels of leisure-time physical activity and healthy lifestyle practices are heavily linked to a reduced risk of periodontitis, improved clinical dental metrics, and better physical fitness/athletic performance. Conversely, high occupational physical activity carries an increased risk.

### Limitation of the studies

Although this is a scoping review and there are no tools to assess the bias present in the selected articles, there are some biases that we must mention. The included human studies are heterogeneous in the design and the measurement of physical activity: questionnaires, self-reports, quantification of VO_2_max, among others. The lack of a standardized exercise type for humans that would allow for the evaluation of, for example, cardiovascular exercise, prevents us from standardizing the results. While most studies demonstrate that it improves inflammatory parameters and protects against bone resorption, there are high-impact studies that could show the opposite. Otherwise, a similar situation occurs with periodontitis, where the clinical parameters are not always the same, and the diagnosis is not based on the latest classification of periodontal diseases. Furthermore, it's noteworthy to mention that while animal studies provide valuable mechanistic information, these models represent a “bubble of reality”. Indeed, human studies consider a heterogeneous population where obesity, smoking, the presence of chronic non-communicable diseases, diet, and other factors could affect both the type of exercise and physical performance itself. Based on questionnaires or self-reports, these variables are not well controlled, making it impossible to draw definitive conclusions. In this context, there is only one controlled clinical trial, which is insufficient to determine whether physical exercise can play a protective role in bone loss caused by periodontitis.

## Conclusion

In experimental models, the physical exercise manages to attenuate inflammation and alveolar bone loss. Most of the selected studies show a marked role of physical exercise as an attenuator of the pro-inflammatory milieu in periodontitis. Nevertheless, randomized clinical trials are needed to determine the true effect of exercise on reducing inflammation and periodontal bone loss.
