# Supplementary material for: Periodontitis and physical activity: a scoping review
Source: Front Oral Health. 2026 Jul 15;7:1786143. doi: 10.3389/froh.2026.1786143 (PMC13416347; doi:10.3389/froh.2026.1786143)
Supplement: Supplementary file 3 [file datasheet1.docx]

**SUPPLEMENTAL FILE 1** Search strategies

Search Strategies for MEDLINE, EMBASE, WEB OF SCIENCE and LILACS databases were the following:

**MEDLINE**

(((((((((((Exercise[MeSH Terms]) OR fitness[MeSH Terms]) OR physical exercise[MeSH Terms]) OR training[MeSH Terms]) OR muscle[MeSH Terms]) OR physical improvement[MeSH Terms]) OR exercise behavior[MeSH Terms]) OR cardiovascular fitness[MeSH Terms]) OR aerobic exercise[MeSH Terms]) OR anaerobic exercise[MeSH Terms]) OR anaerobic resistance[MeSH Terms])AND (((((((((periodontitis[MeSH Terms]) OR chronic periodontitis[MeSH Terms]) OR experimental periodontitis) OR ligature periodontitis) OR oral gavage periodontitis[MeSH Terms]) OR experimental periodontitis model) OR bone resorption[MeSH Terms]) OR periodontal bone resorption) OR bone destruction[MeSH Terms]) AND ((((Human[MeSH Terms]) OR human samples[MeSH Terms]) OR human periodontium[MeSH Terms]) OR periodontal biopsies[MeSH Terms])))).

**EMBASE**

('periodontitis'OR ‘periodontitis model'/exp OR 't oral gavage'/exp OR ‘ligature method'/exp OR ‘bone resorption'OR ‘bone destruction'OR 'inflammation' OR ‘periodontal bacteria'OR ‘periodontal pathogen'OR ‘oral bacteria' OR ‘oral pathogen' OR ‘keystone pathogen' OR 'pathobiont' OR 'microbiota' OR 'dysbiosis' OR ‘dysbiotic microbiota'/exp OR ‘subgingival microbiota' OR ‘periodontal microbiota' OR ‘bacteria detection'/exp OR ‘bacteria quantification' OR ‘bacteria identification') AND ('excersive'/exp OR 'fitness'OR ‘physical exercise'OR ‘aerobic exercise'OR ‘anaerobic exercise'OR ‘aerobic capacity'OR ‘anaerobic capacity'OR ‘cardiovascular health’AND [embase]/lim

**WEB OF SCIENCE**

#1 TS=(Periodontitis OR Pathogen OR Experimental periodontitis OR Bacteria OR keystone OR pathobiont)

#2 TS=(exercise OR fitness OR physical activity)

#3 #1 AND #2}

**LILACS**

“PERIODONTITIS" OR “EJERCICIO FÍSICO" [Palabras] or “ENFERMEDAD PERIODONTAL" [Palabras] and ( “RENDIMIENTO FÍSICO" ) [Palabras]
